# Intestinal Barrier Disturbances in Haemodialysis Patients: Mechanisms, Consequences, and Therapeutic Options

**DOI:** 10.1155/2017/5765417

**Published:** 2017-01-17

**Authors:** D. S. March, M. P. M. Graham-Brown, C. M. Stover, N. C. Bishop, J. O. Burton

**Affiliations:** ^1^Department of Infection Immunity and Inflammation, College of Medicine, Biological Sciences and Psychology, University of Leicester, Leicester, UK; ^2^John Walls Renal Unit, University Hospitals Leicester NHS Trust, Leicester, UK; ^3^National Centre for Sport and Exercise Medicine and School of Sport, Exercise and Health Sciences, Loughborough University, Loughborough, UK; ^4^Department of Cardiovascular Sciences, University of Leicester and NIHR Leicester Cardiovascular Biomedical Research Unit, Glenfield Hospital Leicester, Leicester, UK

## Abstract

There is accumulating evidence that the intestinal barrier and the microbiota may play a role in the systemic inflammation present in HD patients. HD patients are subject to a number of unique factors, some related to the HD process and others simply to the uraemic milieu but with common characteristic that they can both alter the intestinal barrier and the microbiota. This review is intended to provide an overview of the current methods for measuring such changes in HD patients, the mechanisms behind these changes, and potential strategies that may mitigate these modifications. Lastly, intradialytic exercise is an increasingly employed intervention in HD patients; however the potential implications that this may have for the intestinal barrier are not known; therefore future research directions are also covered.

## 1. Introduction

Cardiovascular disease is the leading cause of mortality in patients with end stage renal disease (ESRD) receiving haemodialysis (HD). Chronic systemic inflammation is a nontraditional cardiovascular risk factor that is present and perpetual in HD patients and is associated with increased morbidity and mortality [1]. Other clinical features and consequences of inflammation in HD patients include frailty, impaired physical functioning, and anaemia as well as an increased risk of hospitalisation and death [2]. Several interrelated factors are all believed to add to the chronic systemic inflammatory environment seen in HD patients. These include (but are not limited to) uraemic toxins, hypervolaemia, hypertension, increased amounts of reactive oxygen species (ROS), reductions in antioxidant defence, compromised protein energy state, increased adipose tissue, infection (including vascular access related, bacterial, and viral infection), and comorbid conditions such as diabetes [1–3].

The gastrointestinal tract is another source of inflammation in HD patients, which is starting to receive increasing interest [4–6]. Significant disruptions to the intestinal barrier are thought to take place in HD patients catalysed by the uraemic environment and importantly the process of HD itself [6–8]. Other factors which are unique to this population, such as changes to the composition of diet and medication, are also thought to contribute [6]. It is thought that such disruptions result in the translocation of endotoxins, digestive enzymes, and gut-produced toxic metabolites into the central circulation [6, 8–10].

These observations were initially based on data reporting the presence of inflammation throughout the gastrointestinal tract (including esophagitis, gastritis, duodenitis, enteritis, and colitis) in a postmortem study of 78 prevalent HD patients [11]. Subsequent studies have shown elevated levels of circulating (gut-derived) endotoxin in HD patients compared to control patients [8, 12, 13], with the highest levels being reported in HD and continuous ambulatory peritoneal dialysis (PD) patients [8, 14], when compared to chronic kidney disease (CKD) patients (stages 3 to 5) [8] and transplant patients [12]. Levels of endotoxin in HD patients have been associated with increased levels of systemic inflammation, as well as increased cardiovascular events and mortality [9, 15, 16].

## 2. The Intestinal Barrier

The intestinal barrier has two primary functions, the first of which is to prevent the transport of harmful substances such as endotoxins, gut-produced antigens, and digestive enzymes from the lumen to the internal environment [17].

**Figure 1:**
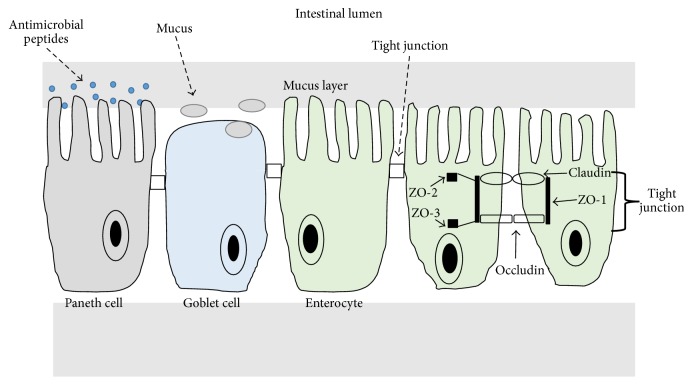
The intestinal barrier.

Secondly, it acts as a semipermeable barrier to allow the selective translocation of essential dietary nutrients, electrolytes, and water from the intestinal lumen into the circulation [17]. This physical intestinal barrier comprises a continuous layer of epithelial cells sealed by intercellular junctional complexes (termed tight junctions [TJs]). These TJs are located on the apical end of the lateral surface of the epithelial cells (Figure 1) [18]. The epithelial stem cells differentiate into four classes of epithelial cells which originate in the crypt and then migrate upwards along the villus axis [19]. The absorptive enterocyte cells comprise 80% of small intestinal epithelial cells, while the goblet cells, the enteroendocrine cells, and the Paneth cells make up the remainder [19]. The Paneth cells are located in the intestinal crypt; their function includes secretion of antimicrobial peptides (e.g., lysozyme and cryptdins or defensins) into the villous crypt, and these are retained in the mucus layer enabling their bactericidal activity to be concentrated near the epithelium [19]. The goblet cells secrete trefoil peptides and mucus which is formed from both glycoproteins and water [20]. This layer of mucus overlays the intestinal epithelium and has an important function in maintaining the intestinal barrier [20, 21].

The TJs are the paracellular barrier and are formed by transmembrane sealing proteins, which include members of the claudin family, occludin, and junctional adhesion molecules. These sealing proteins (claudin and occludin) interact with cytoplasmic proteins including zonula occludens proteins (ZO-1, ZO-2, and ZO-3), from the adjacent cell, and are linked to the cytoskeleton (Figure 1). Claudin and occludin formation at the TJ are regulated by various signalling pathways, which results in the phosphorylation of phosphates, kinases, and signalling molecules [22].

The transcellular barrier is controlled by the enterocyte cells only allowing the permeation of solutes predominantly regulated by specific transporters for amino acids, electrolytes, short-chain fatty acids, and sugars [23]. The TJs function as a selective semipermeable barrier that allows the passage of ions and solutes through the paracellular space while prohibiting the translocation of endotoxin, microbial fragments, and gut-produced toxins into the circulation [17].

## 3. Consequences of Changes in Intestinal Barrier Status in Haemodialysis Patients

The intestines contain large amounts of Gram-negative bacteria [34], which contain complex lipopolysaccharides (LPS) termed endotoxin within the outer cell wall [34, 35]. The terms LPS and endotoxin are frequently used interchangeably. Endotoxin is the natural form of LPS which occurs within the outer wall of Gram-negative bacteria, while LPS refers to the purified form which is used as measurement standard in endotoxin detection assays [36]. Endotoxins have a molecular weight varying from 10 to a 1000 kDa and are comprised of a lipid A antigen attached to a carbohydrate core and polysaccharide O antigen [37].

It is normal for small amounts of endotoxins to cross the intestinal barrier into the circulation; they are regulated and removed by reticuloendothelial cells, phagocytes in the liver [38], mesenteric lymph nodes [39], anti-endotoxin antibodies [38, 40, 41], and lipoproteins [42]. This low level of endotoxin crossing the intestinal barrier allows an interaction between the luminal contents and the mucosal immune system, thus preventing an excessive immune response when an antigen is delivered from the gut into the portal circulation; this allows a state of antigen specific tolerance known as oral tolerance [40]. These bacterial components are harmless when they are restricted to the gut [41] but are highly toxic if they cross the intestinal barrier and enter the circulation [38]. The translocation of endotoxin across the intestinal barrier either through the transcellular or paracellular route induces inflammation through its attachment to toll-like receptor-4 (TLR-4), and cluster of differentiation-14 (CD-14) receptors expressed on the surface of human monocytes and macrophages. This process triggers the release of proinflammatory cytokines such as tumour necrosis factor-alpha (TNF-*α*), interferon-*α* (IFN-*α*), and interleukin-6 (IL-6), via the transcriptional nuclear factor k-light chain enhancer of activated B cells (NF-*κ*b) [43], which results in an inflammatory environment. The influx of these proinflammatory cytokines can further exacerbate the translocation of endotoxin through disruption in the transcellular tight junction proteins (claudin and occludin) promoting systemic inflammation [44, 45]. Not only are disturbances to the intestinal barrier a source of inflammation in HD patients through the process of endotoxin translocation, but they also may contribute to the high number of infections reported in this patient population [46]. The translocation of endotoxins and luminal toxins could place additional stress on the immune system of HD patients and therefore could be less able to defend against other potential pathogens [47]. These theories are yet to be confirmed however.

Increased circulatory concentration of endotoxin is a strong risk factor for the development of atherosclerosis in the general population [48] and may be related to the degree of both atherosclerosis and inflammation in PD patients [14]. This suggests that the translocation of endotoxin and the consequential development of atherosclerosis may be one element responsible for the increased cardiovascular risk in HD patients. Furthermore, changes to the intestinal barrier, the translocation of endotoxin, and diffusion of urea into the intestinal lumen may also increase the circulating levels of toxic metabolites such as *p*-cresyl sulphate (PC) and indoxyl sulphate (IS), which are independent predictors of cardiovascular disease and mortality [49–52]. Intriguingly, PC and IS have also been shown to be predictors of CKD progression [53].

## 4. Haemodialysis Related Factors

### 4.1. The Haemodialysis Process

Levels of circulating endotoxin are significantly elevated in the circulation of HD patients compared to healthy controls [8, 12, 13] (Table 1). Studies also suggest that initiation of HD results in a significant increase in endotoxin levels (from 0.13 ± 0.3 EU/mL to 0.34 ± 0.42 EU/mL) [8]. This is supported by Terawaki et al. [33], who observed increased concentrations of circulatory endotoxin in patients at the end of HD compared to the beginning, though differences did not reach statistical significance [33]. Conversely, Markum et al. [27] found no change in endotoxin levels before and immediately following HD, although this may be explained by a small sample size and the timing of samples. It is known that HD in combination with ultrafiltration results in a systemic haemodynamic perturbation with significant splanchnic ischaemia and hypoxia [7, 8, 54]. This effect is accentuated in patients who have large ultrafiltration volumes during HD [26]. This in turn results in disturbances to the intestinal barrier and significant rises in circulating endotoxin [8, 26]. The vascular architecture of the intestines makes them particularly sensitive to low oxygen environments (both ischaemia and hypoxia) [55], for example, when blood flow is shunted away from the splanchnic region during HD. Intriguingly, it is known that body temperature increases during HD [56] and, given that rises in core temperature have been shown to disturb the intestinal barrier [57–59], it is likely that this increase in blood temperature during HD may have direct implications for the translocation of endotoxin.

### 4.2. Haemodialysis Frequency, Ultrafiltration, and Dialysate

A cross-sectional study showed thrice weekly (conventional) HD was associated with a significantly higher circulating endotoxin level compared to both nocturnal and short daily HD [26] (Table 1). This may be explained by the lower ultrafiltration volumes and rates in the nonconventional HD groups, resulting in lower splanchnic ischaemia and hypoxia and less disturbance to the intestinal barrier [26]. This study concluded that less aggressive ultrafiltration may be a useful strategy to reduce intestinal barrier disturbances in HD patients. These observations may be explained by the findings of another study [60], which postulated that smaller intradialytic fluid gains and the resultant decrease in gut oedema result in reduced endotoxin translocation. Gut oedema has been shown to perturb the intestinal barrier and increase circulating endotoxin levels in chronic heart failure patients [61].

It has previously been shown that cooling the dialysate during treatment has a protective effect on vulnerable vascular beds such as the heart [62, 63] and the brain [64]. This is believed to be driven by an improvement in systemic vascular resistance [62]. It is therefore entirely possible that cooled dialysate can have a protective effect on the intestine. As previously mentioned, body temperature increases during HD [56]; cooled dialysate may protect the intestine not only through haemodynamic mechanisms but also through the direct effect of lower temperature which may itself abrogate any HD induced changes to the intestinal barrier [58].

There is an increasing interest in how changes to the microbiota in HD patients result in the production of toxic metabolites such as PC and IS [5, 6]. By modifying either the dialysis frequency or ultrafiltration (or both) to induce lower splanchnic ischaemia/hypoxia and resultant lower intestinal barrier disturbances, this may have subsequent effects on the concentrations of circulating PC and IS. The investigation of such an effect is clearly an important future research direction.

### 4.3. Intradialytic Exercise

Unlike patients with chronic cardiac and respiratory diseases, exercise is not a commonly used therapeutic intervention in HD patients, despite there being a number of potential benefits [65, 66]. Exercise interventions that occur during HD sessions (intradialytic exercise) are being increasingly employed at HD units [67–69]. However, the potential of intradialytic exercise to modify the intestinal barrier is yet to be investigated. Strenuous exercise in the general population has been shown to disturb the intestinal barrier [34, 59, 70, 71] and results in elevated levels of circulating endotoxin [35]. During exercise blood is redistributed towards the exercising muscles and the skin for heat dissipation and towards tissues with increased metabolic activity such as the heart, lungs, and brain. This leads to a subsequent reduction in splanchnic blood flow [72, 73] and raised core temperature and an increase in ROS [58, 59, 74], all of which have the ability to disturb the intestinal barrier and result in the translocation of endotoxin into the central circulation [38, 59]. Though not specifically studied, it is likely that these physiological changes will be amplified during intradialytic exercise [67] as a result of reductions in blood volume and pressure through the process of ultrafiltration. In addition, the HD membrane is known to increase the production of ROS [75, 76].

In the general population, regular disturbances to the intestinal barrier during exercise may be advantageous through the production of endotoxin antibodies and enhanced clearance via the reticuloendothelial system [35], a form of self-immunisation. Whether regular intradialytic exercise would have such an effect in HD patients is uncertain due to the more persistent change to their intestinal barrier. There is, however, evidence in HD patients that a six-month programme of intradialytic exercise reduced the proportion of monocytes classified as the intermediate subset (CD-14++CD-16+) [77]. This subset is associated with secretion of proinflammatory cytokines in response to LPS-stimulation and a high expression of TLR-4 [78, 79]. Furthermore, elevations in circulating intermediate monocytes are associated with an increased cardiovascular risk in HD patients [80]. As CD-14 plays a role in endotoxin neutralisation, this may be a potential mechanism by which circulating endotoxin is reduced, although clearly further work is needed to corroborate this.

Intradialytic exercise has previously been shown to increase aerobic capacity in HD patients [68] and it would appear that more physically active individuals in the general population with higher aerobic capacity have lower circulating endotoxin levels [81, 82]. This could contribute to some of the anti-inflammatory effects associated with regular physical activity [81]. Whether these data extrapolate to the HD population remains to be seen but it does seem plausible that increasing aerobic fitness and physical activity levels through intradialytic exercise in HD patients might lead to lower circulating endotoxin concentrations and improved outcomes.

Regular exercise training programmes in HD patients may induce favourable changes at a cellular level. Heat Shock Proteins (HSPs) are intracellular molecular chaperones that play a role in protein synthesis and cell maintenance [83]. HSPs also have a repair function to enhance cell survival when challenged by stress and play a regulatory role by modulating protein transcription activity [84]. Upregulated HSP levels have been observed following exercise in healthy individuals [85, 86], and it is increasingly acknowledged that they play a role in protecting the intestinal barrier against stress induced changes [57, 59, 87, 88]. Whether this is the case in HD patients is currently unclear. The strategy of using nutritional interventions (e.g., glutamine, bovine colostrum, and zinc carnosine) to directly target the expression of HSP and protect the intestinal barrier against exercise induced disturbances has recently proven to be effective [57, 59, 88]. Moreover, there is evidence that probiotic supplementation may upregulate HSP expression in vitro [89]. Whether these nutritional therapies could be successfully implemented in HD patients to modify the microbiota and prevent changes to the intestinal barrier is unclear, but they certainly have therapeutic potential and therefore clearly warrant further attention.

## 5. Non-Haemodialysis Related Factors

### 5.1. Uraemia

It has been hypothesised that disturbances in the intestinal barrier are exacerbated in patients with ESRD through diffusion of urea into the gut lumen. Urea is metabolised by gut bacterial urease to ammonia (CO[NH_2_]2 + H_2_O → CO_2_ + 2NH_3_) which in turn is hydrolysed to caustic ammonium hydroxide (NH_3_ + H_2_O → NH_4_OH), breaking down the intestinal barrier [90]. This was elegantly shown in two previous studies [91, 92]. T84 cells were incubated in media containing pre-HD plasma, post-HD plasma, and plasma from healthy controls [91]. Exposure of the cells to the pre-HD plasma resulted in a marked decrease in transepithelial electrical resistance (TER), indicating an increase in permeability [91]. This was paralleled by reduced expression of the tight junction forming proteins (claudin and occludin) [91]. Interestingly the intestinal dysfunction was significantly less in the cells exposed to the post-HD, rather than the pre-HD, plasma [91]. It was hypothesised that it was the presence of unidentified, dialysable product(s) in uraemic plasma which impaired intestinal barrier function. In a follow-up study [92], T84 cells were incubated with clinically relevant concentrations of urea, with the authors reporting an incremental, concentration dependent fall in TER and reductions in the expression of claudin, occludin, and ZO-1. When the T84 cells were incubated with urease to simulate the microbial environment, reduction in the expression of tight junction proteins was augmented [92]. These studies demonstrated that urea is at least partly responsible for the disturbance to the intestinal barrier, in the absence of HD induced barrier changes (ischaemia/hypoxia, ROS). This is also highlighted by other findings [93], which reported no significant differences in plasma D-lactate (a marker of intestinal barrier changes) between patients receiving HD and those choosing conservative treatment [93]. In addition, McIntyre et al. [8] demonstrated increasing circulating endotoxin levels with increasing CKD stage, confirming that the higher the blood urea concentration, the greater the effect on the intestinal barrier.

### 5.2. The Microbiota and Diet

Another important factor that could disrupt the intestinal barrier in ESRD patients is changes in the composition of the microbiota. The intestine is inhabited by some 10^14^ commensal bacteria which provide protection against bacterial pathogens by marinating the intestinal barrier [94]. It is believed that the mechanisms behind the protective effect of the microbiota on the intestinal barrier include stimulating epithelial cell turnover, promoting mucus secretion, upregulation of antimicrobial peptides, restoring tight junction protein structure, and production of short-chain fatty acids and bactericidal proteins [94]. Dietary restrictions in ESRD patients significantly alter the microbiota of the intestinal tract [6]. Restrictions in fruit and vegetable consumption (sources of potassium) and diets low in symbiont-rich cheese/yogurt promote a predominance of bacteria that produce toxic metabolites [50, 95]. These toxic metabolites, which include the aforementioned PC and IS, are produced directly from amino acid bacterial fermentation [49–52]. There is some evidence that dietary interventions such as high-fibre diets may reduce the circulating concentration of PC and IS [96] and some inflammatory markers [97] in HD patients. It is thought that this effect is mediated by the production of short-chain fatty acids, which are used by the epithelial cells as energy [6]; whether this confers a direct upregulation of the intestinal barrier is unclear. It is worth mentioning that these dietary fibre interventional studies contain small sample sizes; consequently larger properly powered investigations with hard end points are necessary. Finally, orally activated charcoal absorbent has shown to partially restore expression of some tight junction proteins and subsequently reduce circulating endotoxin levels in an animal model [98]. However, perhaps unsurprisingly in two randomised controlled trials [99, 100] in CKD patients, orally activated charcoal absorbent proved ineffective in slowing CKD progression; these findings suggest it may be inefficacious in improving outcomes in HD patients. Although outside the scope of this review per se, these results [99, 100] highlight the current uncertainty within the literature of the role that intestinal barrier and microbiota changes have to play in CKD progression and outcomes in both CKD and HD patients.

### 5.3. Medication

The impact of medication on the intestinal barrier, circulating endotoxins, and the microbiota in HD patients is not well understood. It has been postulated that the frequent use of antibiotics and phosphate binders in patients with ESRD may alter the microbiota [5, 6] and consequently compromise the intestinal barrier. Interestingly, though, a study by Sun et al. [31] showed that patients taking sevelamer hydrochloride (SH) had lower circulating endotoxin concentrations, which was followed by another investigation demonstrating that 3 months of SH ingestion proved effective in actually lowering circulatory endotoxin [28]. It has been postulated that SH may have a direct effect in the intestinal tract by binding to endotoxins although the precise mechanism for this effect is yet to be elucidated. Whether other phosphate binders have similar effects is unclear. Iron replacement therapy (IRT) is commonly prescribed in HD patients primarily to increase the efficacy of recombinant erythropoietin therapy [101]; administering iron intravenously will have secondary effects on the intestinal barrier and microbiota. There are conflicting results on the effects of iron administration, with some human and animal studies reporting increased dysbiosis of the microbiota [102–104]; contrarily, in a randomised controlled trial in inflammatory bowel disease patients, intravenous IRT resulted in shifts in microbiota diversity [105] intimating that it may negate microbiota imbalance. The differences in these results may be explained by the initial presence or absence of iron deficiency or anaemia in the studied patients or animals and possibly the route of administration (oral versus intravenous). IRT in HD patients may increase microbiota diversity in the presence of iron deficiency or anaemia; whether this will have consequences for the intestinal barrier is not known, although an increased diversity is generally viewed as a beneficial physiological change.

## 6. Other Considerations: The Assessment of Intestinal Barrier Status in Haemodialysis Patients

The measurement of circulating endotoxin levels has been the primary method of assessing intestinal barrier status in HD patients. The Limulus Amoebocyte Lysate (LAL) assay has been the assay of choice for detecting circulating endotoxin in this patient population [8, 9, 15, 27] (Table 1). Endotoxin levels have previously been expressed either in weight or in endotoxin units (EU) (Table 1); however it has been recommended that levels should be reported as EU rather than using weight as a unit of measurement [106]. Consistency of unit reporting will allow study results to be compared more easily (Table 1).

There is clearly a large variation in previously reported concentrations of endotoxin in HD patients as can be seen in Table 1. This variation may be explained by the timing of the blood samples (either before, during, or after HD), with it not being clear in some studies when the sample for endotoxin analysis was obtained in relation to the participants HD regime [8, 9, 12, 16]. There are a number of substances contained in uraemic plasma that could interfere with the detection of endotoxin by the LAL assay [107], and these in turn could vary throughout the HD process. For example, beta D-glucan can be introduced to the circulation of patients through the use of cellulose membranes during HD [108]. It is possible that the LAL assay may not discriminate between endotoxin and beta D-glucan [27] resulting in false positive results, which has been supported by recent data [109]. Concerns relating to differing reporting units and possible interference of the LAL assay with contaminants (e.g., beta D-glucan) must be taken into account when interpreting and comparing data.

Endotoxin can also be introduced exogenously as a consequence of biofilm formation in the HD machine or in tunnelled central venous catheters [37, 110], possibly resulting in an influx of endotoxin during and immediately following treatment. Additionally, endotoxin may enter the circulation through contaminated dialysate or water. Taken together, the potential sources of endotoxin within HD patients are vast and may not always indicate changes in the integrity of the intestinal barrier of HD patients.

More recent investigations have employed gut bacterial DNA fragments as a method to assess intestinal disturbances in HD patients [10, 93, 111]. When detecting bacterial DNA it is not always possible to confirm the source (intestinal or non-intestinal), and so studies cannot always characterise the bacterial species present in HD patients. Alternate investigations have shown that plasma intestinal-fatty acid binding protein (I-FABP) is a marker of intestinal “damage” and associates with intestinal ischaemia [112]. The use of plasma I-FABP to assess intestinal barrier status in HD patients may hold promise. However, it is probable that a combination of these markers may allow a more advanced assessment of the intestinal barrier status of HD patients, as has been previously suggested [20].

## 7. Conclusion

The importance of changes to the microbiota as a result of a number of factors that are unique to HD patients has received particular recent attention within the literature. The consequences of modulations to the intestinal barrier and the microbiota are believed to contribute towards systemic inflammation in this patient population. Therapeutic interventions that target the intestinal barrier and the microbiota are promising areas of future research in HD patients.

**Figure 2:**
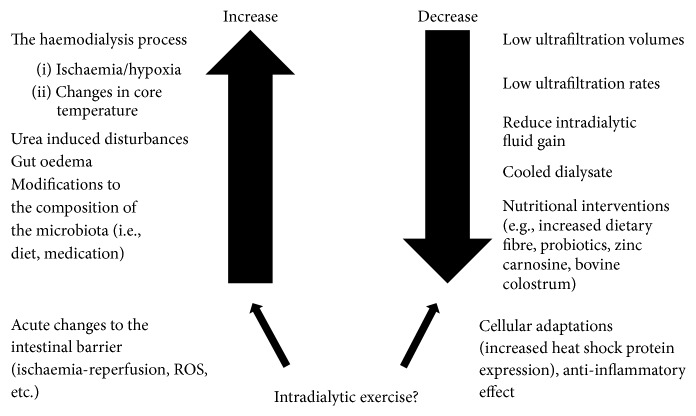
Modifications to the intestinal barrier in HD patients.

It is clear that there are a number of factors which are distinct to HD patients that can disturb the intestinal barrier, including processes related to, and independent of the HD process (see Figure 2). Some strategies have been shown to reduce circulating endotoxin levels in HD patients and in turn there are nutritional strategies that have been shown to modify the intestinal barrier. Their efficacy in HD patients is yet to be shown. It is certainly possible that exercise during HD may have an anti-inflammatory effect through changes in circulating endotoxin or on the intestinal barrier directly (or both), though this is yet to be determined.

## Figures and Tables

**Table 1:** Studies reporting circulating endotoxin in HD patients.

Study	Patients (*n*)	Detection method	HD patients endotoxin concentrations (reported as mean ± SD or median-range)	Control group endotoxin concentrations	Timing of Blood Samples (before, during, or after HD)
[15]	50	LAL assay (gel clot)	76.30 ± 42.09 pg/mL	N/A	After HD
[9]	306	LAL assay (chromogenic)	2.31 ± 3.10 EU/mL	N/A	Not reported
[24]	50	LAL assay (chromogenic)	0.69 ± 0.30 EU/mL	0.04 ± 0.01 EU/ml (*n* = 15) [25].	Before HD and after HD
[16]	25	LAL assay (chromogenic)	0.302 ± 0.083 EU/mL and 0.209 ± 0.044 EU/mL (before and after 4 weeks of conversion to ultrapure dialysate)	N/A	Not reported
[26]	86	LAL assay (chromogenic)	0.66 ± 0.29 EU/mL and 0.08 ± 0.04 EU/mL (for conventional and nocturnal HD patients)	N/A	Before HD
[27]	10	LAL assay (chromogenic)	5.4 pg/dL before HD and 4.63 pg/dL after HD	N/A	Before HD and after HD
[8]	66	LAL assay (chromogenic)	0.64 EU/mL	~0.045 EU/ml (*n* = 14)	Not reported
[28]	59	LAL assay (chromogenic)	0.58 EU/mL (0.51–0.60) and 0.60 EU/mL (0.51–0.63) (before randomisation to sevelamer hydrochloride or calcium acetate)	N/A	Before HD
[12]	31	LAL assay (chromogenic)	40 ± 4.7 ng/L	7 ± 0.6 ng/L (*n* = 99)	Not reported
[29]	20	LAL assay (gel clot)	0.5 to 5.0 pg/mL in 18 samples	N/A	During febrile episodes on HD
[30]	211	LAL assay (chromogenic)	0.65 (0.43–1.16) EU/mL	N/A	Before HD
[31]	46	LAL assay (chromogenic)	0.23 ± 0.01 and 0.30 ± 0.01 (patients taking sevelamer and those not)	N/A	Before HD
[32]	58	LAL assay (turbidimetric kinetic)	0.17 ± 0.11, 0.28 ± 0.15, 0.45 ± 0.16 EU units	N/A	Before HD and after HD
[33]	17	Laser scattering photometry	0.23 EU/mL start of HD and 0.37 EU/mL end of HD	N/A	During HD
[13]	87	LAL assay (chromogenic)	Significant endotoxaemia detected in 6/87 HD patients (27.67 ± 23.56 pg/mL)	5.3 ± 1.1 pg/mL (*n* = 22)	During HD
